# Evaluation of More Stamina, a Mobile App for Fatigue Management in Persons with Multiple Sclerosis: Protocol for a Feasibility, Acceptability, and Usability Study

**DOI:** 10.2196/18196

**Published:** 2020-08-04

**Authors:** Guido Giunti, Octavio Rivera-Romero, Jan Kool, Jens Bansi, Jose Luis Sevillano, Anabel Granja-Dominguez, Guillermo Izquierdo-Ayuso, Diego Giunta

**Affiliations:** 1 University of Oulu Oulu Finland; 2 Universidad de Sevilla Sevilla Spain; 3 Kliniken Valens Valens Switzerland; 4 Hospital Nisa Sevilla Sevilla Spain; 5 Hospital Italiano de Buenos Aires Buenos Aires Argentina

**Keywords:** multiple sclerosis, mHealth, fatigue, fatigue management, apps, gamification, user-centered design, usability, physical activity, eHealth, chronic conditions

## Abstract

**Background:**

Multiple sclerosis (MS) is one of the world’s most common neurologic disorders leading to severe disability in young adults. MS-related fatigue directly impacts on the quality of life and activity levels of people with MS. Self-management strategies are used to support them in the care of their health. Mobile health (mHealth) solutions can offer tools to help symptom management. Following a user-centered design and evidence-based process, an mHealth solution called More Stamina was created to help persons with MS manage their fatigue.

**Objective:**

The overall study aims are to explore the feasibility, acceptability, and usability of More Stamina, a mobile app for fatigue self-management for persons with MS.

**Methods:**

A mixed-methods, multicenter study will be used to assess the feasibility, acceptability, and usability of More Stamina. The study will take place during the third and fourth quarters of 2020 (Q3-Q4 2020) in 3 locations: Argentina, Spain, and Switzerland. A longitudinal cohort study will take place, and think-aloud protocols, open-ended interviews, and short answer questionnaires will be used. Persons with MS will be recruited from the different locations. This study seeks to enroll at least 20 patients that meet the criteria from each site for the longitudinal cohort study (total n=60).

**Results:**

Ethical approval has been granted in Argentina and is pending in Spain and Switzerland. Outcomes will be published in peer-reviewed medical journals and presented at international conferences.

**Conclusions:**

Findings from this study will be used to help understand the role that mHealth can play in fatigue management in MS.

**Trial Registration:**

ClinicalTrials.gov NCT04244214; https://clinicaltrials.gov/ct2/show/NCT04244214

**International Registered Report Identifier (IRRID):**

PRR1-10.2196/18196

## Introduction

### Background

Multiple sclerosis (MS) is one of the world’s most common neurologic disorders leading to severe disability in young adults. More than 2.3 million people live with MS in the world, with higher incidences in Northern Europe and in temperate climates [1]. MS is a chronic condition with high self-management needs [2] that require significant support [3-5] since the majority of self-management occurs at home [6]. MS symptoms range from simple visual disturbances and altered sensation to severe fatigue and cognitive problems with mobility issues [2]. Persons with MS can also go through stretches of periods in which symptoms worsen, called “attacks” or “relapses” [1,2]. Fatigue is the most common and disabling symptom of MS, affecting up to 80% of patients [7-12]. There seems to be some association between MS fatigue and environmental conditions such as the time of the day and weather [13] but it is still unknown how much of an influence these factors have. MS fatigue has a huge impact on quality of life [8,9] and socioeconomic status for persons with MS and is the major reason for early retirement [14]. As a result of MS, persons with MS are typically less active [15] and have reduced levels of physical activity [16-18].

Mobile health (mHealth) is the delivery of health care or health care–related services through the use of portable devices [19]. The use of mHealth software apps has grown in recent years, to the point where commercial app stores hold thousands of health care–related apps [20]. Several digital and remote communication technology apps have been developed for MS clinical monitoring and management, to complement traditional clinical approaches [21]. Wearable devices, including actigraphy, gyroscopes, and body temperature or heart rate monitors, have been used to obtain a more comprehensive assessment of different body functions and to monitor disability in persons with MS [22]. A recent review [21] showed that the use of apps for MS as complements to traditional in-clinic care can improve outcomes and increase access to care, disease information, and support. The use of mHealth could help persons with MS to be more active in their self-management, for example, by tracking adherence to treatment, changes in bladder and bowel habits, and activity and mood.

However, digital health technologies have low adoption rates by patients with chronic diseases, in part explained by an inappropriate fit of these technologies with patients’ daily lives and the high patient burden associated with digital self-monitoring [23]. Studies regarding the attitudes of persons with MS toward using smartphone apps highlight the benefits of tailoring apps to specific patient needs [24,25]. Further, involving patients and health care professionals in the design of technology is a need that has been often raised [26-32] and could be helpful in increasing adoption rates [33-35].

In previous studies, we explored available mHealth apps for persons with MS and found that only a handful exist [31,36]. This prompted the user-centered design (UCD) process of an mHealth solution specifically tailored for this target population called More Stamina.

### User-Centered Design

UCD is a design philosophy that places the needs and characteristics of end users in the center of software design and development [37-39]. Through the use of UCD, solutions that are specific to the characteristics of the intended users are designed with higher acceptance and fewer user errors [38-40]. The overall process of UCD comprises the specification of the context of use (understand users and their characteristics and environment), specification of the requirements (identify the granular requirements and needs), production of solutions (start an iterative process of design and development), and evaluation (testing to find critical feedback on the product) [37,41].

The evaluation of usability entails a wide array of methodologies that vary in terms of research design, complexity, cost, and duration [42]. Different methods can be used to evaluate a system design on its usability, such as expert-based inspections and user-based testing [43]. Following UCD ensures that mHealth apps are more likely to meet end user needs and expectations [44,45].

### More Stamina

**Figure 1:**
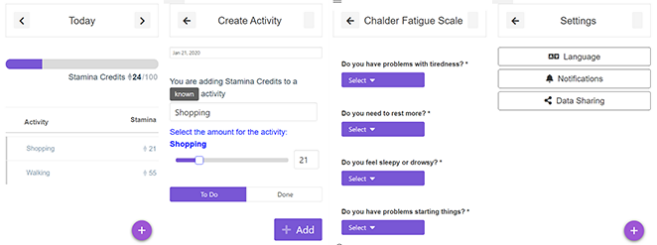
More Stamina screenshots.

More Stamina is a gamified task organization tool designed to help persons with MS manage their energy, to minimize the impact of fatigue on their daily life. The tool acts as a to-do list where users can input the tasks they want to accomplish that day in a simple manner (Figure 1). More Stamina is currently available in English, Spanish, Finnish, and German.

The overall concept is that a person’s energy is represented through a visual metaphor (progress bar) and a symbolic unit that quantifies the amount of estimated effort a given activity might take (Stamina Credits). Users start their day with 100 Stamina Credits and assign them to new activities for that day. As users go through their day and complete the activities, they mark them as done, and the system prompts them to assess whether their effort was underestimated, overestimated, or properly estimated. More Stamina keeps track of these answers as data points and starts analyzing and creating a trend for each activity (eg, “shopping”). Repeated use of More Stamina allows it to learn about the user’s habits, and once sufficient information is gathered on “shopping,” a recommendation feature starts reminding users of their own tendencies.

The app logs user activity through the in-built sensors of the device and is able to monitor the number of steps, walking pace, distances, and GPS positioning. Patient-reported outcomes are available and optional in More Stamina. Standardized tools such as the Fatigue Severity Scale [46] and Chalder Fatigue Scale [47] are loaded into the system. Usage statistics are gathered locally for each added activity to keep track and collect assessments; the user can choose to share these statistics to a secure server for analysis. Users have control as to which information to disclose and with whom, whether it’s personal, clinical, or treatment-related. Additionally, they can opt-in to send de-identified information for research purposes.

### Current Status

The More Stamina app was created following the UCD approach through iterative development, allowing continuous improvement of the app. The progress of the solution through the different phases of the UCD process is reported in our previous studies. The state of the practice of health apps for MS was studied through a systematic app review [31,36]. The needs, barriers, and facilitators of mHealth apps for persons with MS were explored using focus groups and interviews [25]. User profiles, or “personas,” were created to aid the design process [25,48], and the design process, prototyping, and initial usability testing have been described [49].

**Figure 2:**
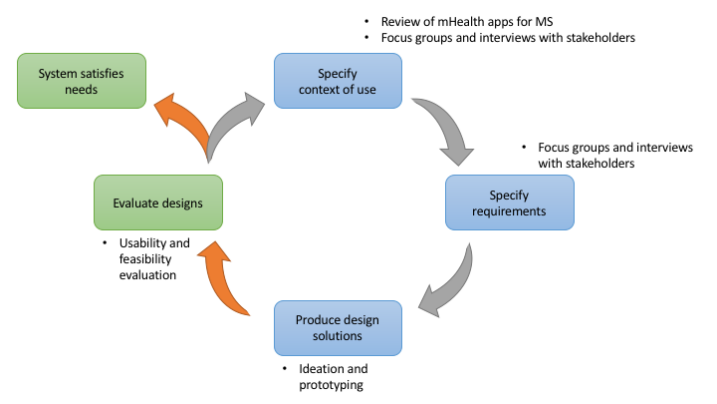
Phases of the user-centered design for the More Stamina app. The green boxes indicate the areas of focus for the current study. mHealth: mobile health; MS: multiple sclerosis.

The work intended in this phase will assess the feasibility, acceptability, and usability of the mHealth solution with persons with MS (see Figure 2).

### Objectives

The overall study aims are to explore the feasibility, acceptability, and usability of More Stamina, a mobile app for fatigue self-management for persons with MS.

The specific objectives are to estimate adherence to the use of More Stamina; estimate the effect of More Stamina adherence on behavior change, measured through changes in the amount of activities and amount of estimated energy per activity; estimate the effect of adherence to the use of More Stamina on the perception of fatigue management; validate the value proposition of More Stamina; identify More Stamina user activity patterns of persons with MS; and identify factors associated with the use of More Stamina.

## Methods

### Study Design

A mixed-methods, multicenter study will be used to assess the feasibility, acceptability, and usability of More Stamina. The study will take place during the third and fourth quarters of 2020 (Q3-Q4 2020) in 3 locations: Argentina, Spain, and Switzerland.

A longitudinal cohort pilot study will be conducted using a series of well-established standardized tools for user-based evaluations and surveys (see Standardized Tools). Participants’ background information regarding health information, quality of life, and familiarity with mobile technologies will be collected. Potential cultural differences will also be explored through qualitative approaches. System usage logs will quantify patient engagement with the system [50].

### Settings

This study is part of a collaborative project between researchers and different institutions across the globe. The work will take place in 3 locations with local teams. Sessions will be conducted by a native language facilitator. Overall study coordination will be carried out by Dr. Guido Giunti from the University of Oulu as part of the More Stamina research project.

#### Argentina

Hospital Italiano de Buenos Aires (HIBA) is a university general hospital in the Autonomous City of Buenos Aires that includes 2 hospitals (Central Hospital and Hospital Italiano de San Justo Agustin Rocca) and 22 primary care centers. This organization has 750 beds, conducts approximately 2.5 million outpatient visits per year, and includes a health maintenance organization that delivers prepaid health care to approximately 150,000 members per year.

The Argentinian local team will be composed of researchers from HIBA.

#### Spain

The Vithas Nisa Sevilla Hospital (VNH) ranks as the number one private center in Andalusia and fourth in all of Spain with a specialized unit aimed at researching and treating MS. This unit is composed of a multidisciplinary team, including neurologists, neuropsychologists, physical therapists, clinical researchers, nurses, and administrative staff.

The Universidad de Sevilla is the main house of learning in the Andalusian province of Spain and provides superior education by means of studies, teaching, and research, as well as the generation, development, and diffusion of knowledge to serve citizens and society.

The Spanish local team will be composed of researchers from the Universidad de Sevilla and VNH.

#### Switzerland

Kliniken Valens is a center specializing in neurological rehabilitation services located in Valens, Switzerland. Kliniken Valens employs a multidisciplinary staff, including neurologists, physiotherapists, occupational therapists, speech therapists, and sports therapists. In 2019, a total of 550 persons with MS were admitted for neurological rehabilitation, of which 70% suffered from fatigue and this was the primary focus of their stay.

The Swiss local team will be composed of researchers from Kliniken Valens and the University of Oulu.

### Recruitment and Sample Size

Persons with MS will be recruited from patient databases at HIBA, VNH, and Kliniken Valens and invited to participate in the study. Inclusion criteria will require each participant to be >18 years old, have received a confirmed MS diagnosis according to McDonalds criteria [51] at least 1 year prior to the study, have none to moderate physical disability (Expanded Disability Status Scale [EDSS] <6.5) at the time of recruitment, have no major cognitive or haptile impairment influencing the ability to use the app, be fluent in one of the languages in which More Stamina is available, and be the owner or user of a compatible smartphone device with internet access.

Exclusion criteria include refusal to participate, cognitive or physical impairment that prevents the use of mobile phones, and inability to attend the follow-up encounters.

This study seeks to enroll at least 20 patients that meet the criteria from each site for the longitudinal cohort study (total n=60). The sample size is an adequate number because of the methods used that provide extensive, detailed data [52].

### Standardized Tools

The following is a description of the standardized tools that are used in this study. Language-appropriate and culturally validated versions will be used accordingly.

#### Expanded Disability Status Scale

The EDSS is a method of quantifying disability in MS and monitoring changes in the level of disability over time. It is widely used in clinical trials and the assessment of persons with MS [53]. The EDSS scale ranges from 0 to 10 in 0.5-unit increments that represent higher levels of disability. Scoring is based on an examination by a neurologist.

#### Quality of Life in Neurological Disorders

Neuro-QoL (Quality of Life in Neurological Disorders) is a measurement system that evaluates and monitors the physical, mental, and social effects experienced by adults and children living with neurological conditions [54].

#### Chalder Fatigue Scale

The Chalder Fatigue Scale is a self-administered questionnaire for measuring the extent and severity of fatigue within both clinical and nonclinical epidemiological populations. Although originally developed to measure the extent of chronic fatigue symptoms within clinical populations, the scale was revised and is now more widely used to measure the severity of “tiredness” rather than just chronic fatigue syndrome [47].

#### eHealth Literacy Scale

The eHealth Literacy Scale (eHeals) is an 8-item scale that tends to measure perceived skills at finding, evaluating, and applying electronic health information to health problems [55]. The instrument has been proven to be a reliable and easy-to-use self-report tool and has been used in some studies. The scale is based on a model that distinguishes between 6 types of literacy skills: traditional literacy, health literacy, information literacy, scientific literacy, computer literacy, and media literacy. Accordingly, the eHeals aims to measure a broad overview of literacy skills, which might make it a potential instrument to assess the effects of electronic health literacy–tailored strategies to deliver online information and apps.

#### System Usability Scale

The System Usability Scale provides a “quick and dirty,” reliable tool for measuring a product’s usability [56]. It consists of a 10-item short questionnaire with 5 response options for respondents, ranging from “Strongly agree” to “Strongly disagree.”

#### Think-Aloud Protocol

Think-aloud is a well-established technique for usability assessment, commonly used to determine users’ thoughts and opinions while they perform a list of specified tasks with a system [57]. A think-aloud protocol asks users to express their immediate thoughts and reactions during their interactions with a system. The sessions are normally recorded, or notes are taken [58]. Minimal intervention from the usability tester assures users’ thought processes are not interrupted except to remind them to keep talking. The focus is on understanding users’ decision-making processes and on how users experience the system in their own words. Only a small sample of users is needed due to the extensive, detailed data it provides [59,60].

To guide the usability evaluation, case scenarios were created for what would be considered as normal everyday user interactions with the More Stamina solution. A panel of neurologists, physiotherapists, and designers validated the different scenarios. The specific scenarios patients will have to perform will consist of (1) completing their user profile, (2) creating a new activity for themselves, (3) managing previously recorded activities, (4) planning for a future activity, and (5) responding to one of the integrated surveys (Multimedia Appendix 1).

Participants will be instructed to assess the More Stamina app using the think-aloud method, stating out loud what they are doing, what thoughts come to their minds, and how are they interacting with the app. The facilitator will record comments and actions of the participants with screen-capturing software and an audio recorder.

#### Open-Ended Interviews

Qualitative inquiries are useful to provide insight into complex and multifaceted experiences of individuals when a rich description is the main goal of the study [61]. Participants of our study will be interviewed with open-ended questions to explore their experiences with the mHealth solution. They will be asked to comment on sections of the system they thought were well designed, to comment on sections that were inadequately designed, and to provide any further comments they might have about system usability.

#### User Behavior

Within More Stamina, Stamina Credits are a numeric continuous variable that ranges from 0 to 100 to represent the estimated effort of an activity. This variable is used to gauge how effective the user is in predicting the effort of each individual activity.

User activity patterns over time will be aggregated per week and described. Adherence will be estimated in each follow-up meeting. Adherence with app use will be estimated using the frequency of weekly use throughout the tracking period.

### Study Flow

Persons with MS from each location will be invited to be part of the study and go through the process of informed consent where they will be briefed about the overall study and their rights as participants (see Ethical Considerations). After providing informed consent, participants will be part of a series of 4 workshops. Prospective monitoring will be carried out for 2 months for all participants at all sites, and daily measurements of More Stamina during that period will be considered.

Encounter 1 will be used to set up user accounts for participants, introduce the More Stamina solution, and install it on their smartphones. Monitoring will begin after the installation of More Stamina. All participants will complete the following standardized tools: EDSS, NeuroQoL, Chalder Fatigue Scale, eHeals, and System Usability Scale.

A subgroup of up to 5 participants per site (total n=15) will be selected for convenience to represent the main themes of this study: time since diagnosis (prolonged or recent), digital health literacy (expert user or beginner user), and disease burden (high or low). This subgroup will go through the think-aloud protocol by performing the specific scenarios and be interviewed about ease, utility, and perceived benefit. The sessions will be digitally audio recorded and video recorded, capturing on-screen navigation on screens.

**Figure 3:**
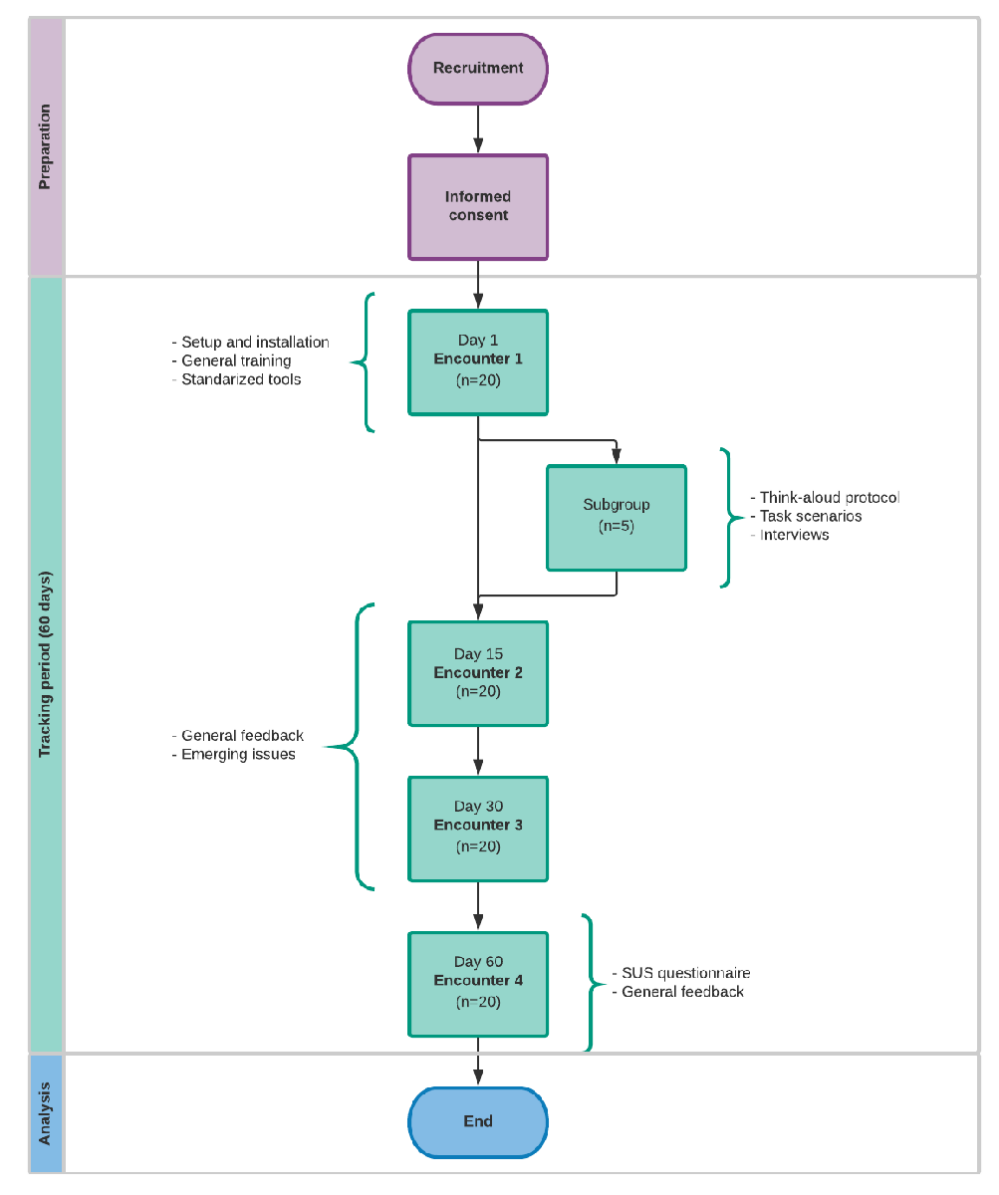
Study flow. SUS: System Usability Scale.

There will be 3 face-to-face follow up meetings: day 15 (Encounter 2), day 30 (Encounter 3), and at the end of the follow-up at day 60 (Encounter 4). In these meetings, feedback will be obtained on the use of the More Stamina app regarding its functionality, usability problems, daily use, and information concerning disability, quality of life, and adherence. The overall study flow that will be replicated in each site is shown in Figure 3.

### Data Analysis

#### Quantitative Analysis

Descriptive statistics will be used to summarize the participants’ background and characteristics. Categorical variables will be presented as absolute and relative frequencies. Continuous variables will be presented as mean and standard deviation or median with interquartile range depending on distribution. A *P* value <5% will be considered statistically significant. Statistical analysis will be performed using STATA v15.

Quantitative analysis will be performed on the data collected through the mHealth solution. A linear regression model will be used to assess the association between the degree of adherence to the app, changes in the amount of activity, and changes in the amount of estimated stamina per activity. The outcome variables will be changes between baseline and final activities, changes in estimated stamina per activity for specific activities selected by the researcher team based on frequency and relevance, and changes in fatigue self-perception. Changes will be calculated considering the baseline measurement and final measurement after the 3-month tracking period. A multivariate linear regression model adjusted for age, sex, and eHEALS will be used. The need for inclusion of other potential confounders will be evaluated.

Participants who are above the median usage of the app will be defined as heavy users. The median is less affected by outliers and skewed data and is usually the preferred measure of central tendency when the distribution is not symmetrical. Potential factors associated with the use of the app will be explored with a univariate logistic regression model.

Qualitative analysis will be performed on the data collected through the think-aloud protocol, individual interviews, and focus groups by using thematic analysis to identify emerging themes [62]. Each transcript will be reviewed and coded independently by 2 researchers. The themes will be combined by agreement of the 2 researchers, involving a third researcher in the event of a disagreement. Major themes and subthemes will be developed via an iterative review process. In order to help ensure the integrity of the content analyses, the guidelines recommended by Shenton [63] will be followed, which include collecting and analyzing data in an iterative process to identify themes and generating an audit trail, among others.

#### Crosscultural Aspects

Cultural contexts influence the way users choose, utilize, and conceptualize products and technology [64]. According to Evers and Day [65], culture is a discernible variable in interface acceptance, and it seems to play some role in perceiving subjective preferences in design [66]. Crosscultural partnerships can positively influence product development, but the use of traditional usability testing techniques in crosscultural settings can be problematic and may produce unexpected or spurious results [67].

Qualitative and quantitative data will be explored, paying attention to potential differences that pertain to cultural and geographical contexts such as traditions, habits, and weather conditions. In order to mitigate the risk of spurious results in usability testing due to crosscultural settings, we will use native speakers who are in a trusted position related to the participants.

### Data Handling

Access to personal information will be restricted to the investigators of the study, health authorities, the Research Ethics Committee, and the monitors and auditors of the study. They will be subject to the duty of secrecy inherent to their profession, when necessary, to verify the data and procedures of the study, but always maintaining the confidentiality of the same according to the current legislation. Participants may exercise their rights of access, rectification, cancellation, and opposition of data according to the European Union General Data Protection Regulations [68].

The information and personal data of the participants will be kept in a completely confidential form with all the rigor of the law. Participants will be asked to not use any names during group discussions. Reports of study results will not include any identifying information. The paper questionnaires used will be digitized. The audio recordings of the group discussions will be typed and kept in secure servers at the University of Oulu. After the transcriptions are finished, the audio recordings will be destroyed. The typed transcripts will remain on password-protected computers, and any hard copy will be kept in a closed filing cabinet. Only members of the research team will be able to listen to the recordings or read the typed versions.

### Ethical Considerations

The ethical research guidelines of the University of Oulu [69] and University of Seville [70] will be followed. Ethical approval will be obtained to ensure that the research is done in accordance with the Declaration of Helsinki and in line with the current local legislations from the respective authorities: HIBA’s Institutional Review Board (Argentina), the Ethics Committee of the Regional Ministry of Health of the Government of Andalusia (Spain), and the Swiss Ethics Committee on Research Involving Humans (Switzerland). The study has been registered in ClinicalTrials.gov with the identifier NCT04244214.

The participants will be informed about the nature of the research project; the reasons for their participation; risks, benefits, and alternatives associated with the research; and their rights as research subjects before agreeing to participate. Steps will be taken to ensure that data gathered from participants will be kept under strict security, anonymity, and privacy.

## Results

The study will take place during the third and last quarters of 2020 (Q3-Q4 2020). Outcomes will be published in peer-reviewed medical journals and presented at international conferences.

## Discussion

### Overview

This protocol presents the work intended to take place to assess the feasibility, acceptability, and usability of a fatigue management mHealth solution for persons with MS.

Persons with MS have a different attitude than other people towards physical activity [18] and are typically less active than healthy persons [15]. The proposed approach of More Stamina for fatigue management is in line with common approaches in energy conservation education programs and fatigue management for MS [71,72]. The goal is to help the patient save energy through the implementation of different strategies such as work simplification or the use of task prioritization.

By tracking and collecting use and contextual information, the present study will also help to understand underlying factors and causes for MS fatigue.

The goal of this pilot study is to further our understanding of the potential issues and challenges that will be used as the foundations for a larger randomized control study.

### Limitations

It is possible that the differences between the patient group who participated in the UCD and the target groups of this study are significant enough that there is a preference mismatch. For example, the importance of fatigue management could vary depending on cultural factors or adherence, or interest could even be radically different depending on economic factors. This is expected, and understanding potential differences is part of the aims of the study. Finally, the nature of the study does not allow the assessment of health outcomes due to the lack of randomized exposure and monitoring period. Such explorations will be the subject of future studies.

## Appendix

Multimedia Appendix 1 Case scenarios.

## Abbreviations

EDSS: Expanded Disability Status Scale
eHeals: eHealth Literacy Scale
HIBA: Hospital Italiano de Buenos Aires
mHealth: mobile health
MS: multiple sclerosis
SUS: System Usability Scale
UCD: user-centered design
VNH: Vithas Nisa Sevilla Hospital
