# Supplementary material for: Evaluation of More Stamina, a Mobile App for Fatigue Management in Persons with Multiple Sclerosis: Protocol for a Feasibility, Acceptability, and Usability Study
Source: JMIR Res Protoc. 2020 Aug 4;9(8):e18196. doi: 10.2196/18196 (PMC7435635; doi:10.2196/18196)
Supplement: Multimedia Appendix 1 [file resprot_v9i8e18196_app1.docx]

## Completing user profile

A friend has told you that More Stamina is worth checking out and you have downloaded the application and installed it from the store. You know that the app uses the information that you enter to provide you with a more personalized experience, so you want to complete your profile. Please complete your profile.

## Creating a new activity

You woke up some time ago and while you are having breakfast you decide that you want to plan your activities for the day. You know that you want to do some shopping, walk the dog and do the laundry. Please add each of these activities in the app.

## Managing previously recorded activities

It is now the middle of the day and you have done one or more of the activities that you have added in your app but you forgot to mark them as complete. Now that you have the time to do that, please mark the activities as completed.

## Planning for a future activity

It is now the evening and you are finishing your day. You realize that there are activities that you know you have to do another day and you want to add them to the app. Please create an activity that will take place 2 days from now.

## Responding a survey

You have just received a notification from the app that tells you that there is an important survey that you need to complete about your condition. You have found a moment in your day that allows you to do the survey without rushing so you sit down and look for the survey. Please answer a survey within the app.
